# Tissue microarray analysis of eIF4E and its downstream effector proteins in human breast cancer

**DOI:** 10.1186/1756-9966-28-5

**Published:** 2009-01-09

**Authors:** Heather E Kleiner, Prasad Krishnan, Jesse Tubbs, Mark Smith, Carol Meschonat, Runhua Shi, Mary Lowery-Nordberg, Patrick Adegboyega, Marcia Unger, James Cardelli, Quyen Chu, J Michael Mathis, John Clifford, Arrigo De Benedetti, Benjamin DL Li

**Affiliations:** 1 Dept. of Pharmacology, Toxicology, and Neuroscience, Breast Cancer Focus Group, Feist-Weiller Cancer Center, Shreveport & LSUHSC-Shreveport, Louisiana, LA, 71130, USA; 2 Dept. Surgical Oncology, Breast Cancer Focus Group, Feist-Weiller Cancer Center, Shreveport & LSUHSC-Shreveport, Louisiana, LA, 71130, USA; 3 Dept. of Medicine, Breast Cancer Focus Group, Feist-Weiller Cancer Center, Shreveport & LSUHSC-Shreveport, Louisiana, LA, 71130, USA; 4 Dept. of Pathology. Breast Cancer Focus Group, Feist-Weiller Cancer Center, Shreveport & LSUHSC-Shreveport, Louisiana, LA, 71130, USA; 5 Dept. of Microbiology & Immunology, Breast Cancer Focus Group, Feist-Weiller Cancer Center, Shreveport & LSUHSC-Shreveport, Louisiana, LA, 71130, USA; 6 Dept. of Cellular Biology & Anatomy, Breast Cancer Focus Group, Feist-Weiller Cancer Center, Shreveport & LSUHSC-Shreveport, Louisiana, LA, 71130, USA; 7 Dept. of Biochemistry & Molecular Biology, Breast Cancer Focus Group, Feist-Weiller Cancer Center, Shreveport & LSUHSC-Shreveport, Louisiana, LA, 71130, USA

## Abstract

**Background:**

Eukaryotic initiation factor 4E (eIF4E) is elevated in many cancers and is a prognostic indicator in breast cancer. Many pro-tumorigenic proteins are selectively translated via eIF4E, including c-Myc, cyclin D1, ornithine decarboxylase (ODC), vascular endothelial growth factor (VEGF) and *Tousled*-like kinase 1B (TLK1B). However, western blot analysis of these factors in human breast cancer has been limited by the availability of fresh frozen tissue and the labor-intensive nature of the multiple assays required. Our goal was to validate whether formalin-fixed, paraffin-embedded tissues arranged in a tissue microarray (TMA) format would be more efficient than the use of fresh-frozen tissue and western blot to test multiple downstream gene products.

**Results:**

Breast tumor TMAs were stained immunohistochemically and quantitated using the ARIOL imaging system. In the TMAs, eIF4E levels correlated strongly with c-Myc, cyclin D1, TLK1B, VEGF, and ODC. Western blot comparisons of eIF4E vs. TLK1B were consistent with the immunohistochemical results. Consistent with our previous western blot results, eIF4E did not correlate with node status, ER, PR, or HER-2/neu.

**Conclusion:**

We conclude that the TMA technique yields similar results as the western blot technique and can be more efficient and thorough in the evaluation of several products downstream of eIF4E.

## Background

Breast cancer remains a major cause of death among women. The American Cancer Society's facts and figures shows that 182,460 new cases of breast cancer will be diagnosed in women in 2008 [1]. The number of deaths due to breast cancer in 2008 is projected to be 40,480. In addition, 1990 men are expected to get breast cancer and 450 to die of it in 2008. There are several risk factors for breast cancer occurrence such as genetic susceptibility, radiation, obesity, and alcohol use. Pathways activated in breast cancer include Eukaryotic Translation Initiation Factor 4E (eIF4E) pathway [2], Phosphatidylinositol-3-kinase(PI3K)-AKT pathway [3], Mitogen-Activated Protein Kinase (MAPK) pathway [4] and the Nuclear factor-kappaB (NFkB) pathway [5]. Our research has focused on the role of the eIF4E in human breast cancer.

### Role of eIF4E in human breast cancer

The eukaryotic translation initiation factor, eIF4E, is a 25-kD cytosolic cap-binding protein that recognizes and binds to the 7-methylguanosine cap in the 5'-untranslated regions (5'-UTR) of mRNAs during the initiation of protein translation (reviewed in [6,7]). eIF4E may be considered the rate-limiting component in translation initiation because it is found in much lower amounts than other translation factors and is activated via mitogenic stimuli (serum, phorbol esters, tumor necrosis factor a, and lipopolysaccharide [6]). Several complex 5'-UTR mRNAs involved in cell division, cell growth, and angiogenesis, are known to be selectively translated via eIF4E, including ornithine decarboxylase (ODC) [8], vascular endothelial growth factor (VEGF) [9], c-Myc [10], cyclin D1 [11], and *Tousled-like kinase *1B (TLK1B) which mediates radioresistance [12]. Furthermore, fibroblast cells transfected with eIF4E develop a malignant phenotype, whereas treatments aimed at inhibiting the level or activity of eIF4E result in inhibition of tumorigenic properties [13]. eIF4E is overexpressed in malignant breast cancer tumor lines MDA-MB-435, MDA-MB-231, and MCF-7, but not in non-tumor cells (MCF-10A) or epithelial cells from the milk of a nursing mother [14]. eIF4E protein expression is also elevated in a variety of human cancers including breast cancer but not in stroma or in benign tissue [13]. Furthermore, eIF4E expression is elevated during hypoxia [15], and at the invasive front in head and neck cancer and in invasive disease [16].

Based on these observations, clinical studies have been conducted to determine the relationship between eIF4E overexpression (quantitated by western blot analysis) and clinical outcome. The results indicated that patients with high eIF4E had a statistically significant higher rate of cancer recurrence (n = 38, p = 0.03 log-rank test) and cancer-related death (n = 38, p = 0.04 log-rank test) compared to those with low eIF4E overexpression in a 40-month follow-up [17]. These results were further supported in a prospective trial, in which patients with high eIF4E overexpression had a shorter disease-free survival (p = 0.004, log-rank test) and higher cancer-related deaths (p = 0.002) compared to those with low eIF4E overexpression. Furthermore, eIF4E protein expression correlated with increased VEGF levels and microvessel density [18]. Significantly, eIF4E expression was independent of ER, PR, HER-2/neu, or node status as determined by Cox proportional hazard model [18,19].

### Fresh-frozen vs formalin-fixed paraffin embedded tissue

As mentioned above, high eIF4E overexpression has been associated with a worse clinical outcome [17]. However, one of the limiting factors in that study was that it required western blot analysis of fresh-frozen tissue. Fresh-frozen tissue is typically scarce, especially in smaller tumors. Furthermore, in order to conduct a multi-institutional study to analyze enough samples for meaningful results, archived specimens will be essential. In addition, the use of paraffin-embedded archived samples would be useful for long-term follow-up. This will enable researchers and clinicians to establish eIF4E as a standard prognostic or diagnostic factor. Additionally, if eIF4E is determined to be a diagnostic factor, it may be used to personalize therapeutic care of the patient.

### Tissue Microarrays

Yang and colleagues recently reported that eIF4E levels were moderately correlated with VEGF and cyclin D1 in a breast cancer TMA [20]. This TMA was obtained from TARP . However, although complete histologic data was available for breast, only limited and incomplete clinical information was available. The goal of our present study was to validate our own in-house TMA's by comparing eIF4E expression with known downstream effector molecules, cyclin D1, c-Myc, VEGF, TLK1B, and ODC. We possess complete clinical information on each specimen, which will allow future TMAs to be constructed for further analysis.

## Materials and methods

### Tissue procurement for western blot analysis

Breast cancer specimens of at least 100 mg were obtained from the tumor core at the time of surgery from each patient per IRB approved protocol. The specimens were verified by the study pathologist to be invasive mammary carcinomas. The specimens were then immediately frozen in liquid nitrogen and stored at minus 70°C for subsequent assay preparations.

### Construction of TMAs

The archived H&E slides used for diagnosis were reviewed by the pathologist on the team for confirmation of diagnosis and selection of appropriate paraffin-embedded tissue blocks for the construction of TMAs. Slides with appropriate tissue of interest were selected and mapped to define representative areas for construction of the TMA blocks using a 1.5 mm punch size. In all, 3 TMA blocks were constructed.

*TMA block 1 *consisted of the following specimens: 5 node positive breast ductal carcinoma, 3 node negative breast ductal carcinoma, 1 ductal carcinoma in-situ, and 1 benign breast tissue. The carcinomas (in-situ carcinoma and the invasive ductal carcinoma cases) were punched in triplicates and the patient-matched controls of corresponding benign breast tissues were punched in duplicates. For slide orientation and as additional tissue control, normal pancreas tissue (punched in duplicate) was also included in each TMA.

*TMA block 2 *consisted of the following specimens: 6 node positive breast ductal carcinoma, 6 node negative breast ductal carcinoma, 2 ductal carcinoma in-situ with matched, 2 benign breast tissues as benign controls from the 2 the patients with ductal carcinoma in-situ, and 1 benign breast tissue from a breast reduction surgery. The invasive carcinomas were punched in triplicates. The in-situ carcinoma cases and the matched benign controls were punched in duplicates.

*TMA block 3 *consisted of the following specimens: 38 invasive ductal carcinoma patients (40 cases punched but 2 had no tumor on the TMA), 3 patients with ductal carcinoma in-situ, and 3 normal breast tissues from breast reduction surgeries.

### Immunohistochemistry

For the immunohistochemical analysis, 5 μm thick sections were cut, warmed to 60°C, de-paraffinized in xylene, and then rehydrated with graded ethanol. This step was followed by antigen exposure for 20 minutes in heated antigen retrieval solution and then the endogenous peroxide activity was inactivated by treating with 0.3% H_2_O_2 _in methanol. The sections were blocked for 20 min in protein block (normal goat serum in PBS, BioGenex), and incubated with primary antibodies against ODC (Sigma #O1136, diluted 1:500); eIF4E (monoclonal, BD Transduction Laboratories, 1:600 dilution), c-Myc (Abcam, ab31426, 1:500 dilution), TLK1B (from De Benedetti [21], 1:700 dilution), VEGF (Ab-3, JH121, NeoMarker-Labvision, 1:60 dilution), and cyclin D1 (Cell Signaling #2926, 1:100 dilution) for 1 h using an automated stainer (BioGenex I6000 Automated Staining System, San Ramon, CA). Samples were rinsed 5 times in washing buffer, and incubated in secondary antibody (MultiLink-BioGenex Super Sensitive Link-Label IHC Detection System) for 30 min. Samples were rinsed 3 times in wash buffer, and then incubated in horseradish peroxidase label (BioGenex) for 15 min. Samples were rinsed 3 times in wash buffer and then incubated in diaminobenzidine (Dako Cytomation Liquid DAB Substrate Chromogen System) for 5 min. Samples were rinsed 3 times in wash buffer and counterstained in hematoxylin (Dako Cytomation Automation Hematoxylin) for 2 min.

### Western Blot

Specimens were analyzed for eIF4E and TLK1B as previously described [22,23]. Briefly protein lysates from each specimen (5–10 μg protein) were separated using 12% denaturing gel Tris-HCL polyacrylamide gel electrophoresis [24]. The proteins were then electroblotted on a nylon membrane (Immun-Blot PVDF, Bio-Rad Laboratory, Hercules, CA) [25]. The membranes were blocked in 3% nonfat milk overnight. Primary incubation of the membranes was carried out using a 1:1000 dilution of monoclonal mouse anti-eIF4E antibody (610270; BD Biosciences, San Jose, CA) or rabbit anti-TLK1B antibody (1:1000 dilution, De Benedetti laboratory). Secondary incubation of the membrane was then carried out using a 1:5000 dilution of goat antimouse or anti-rabbit IgG tagged with horseradish peroxidase. The blot was developed using Opti-4CN substrate kit (Bio-Rad Laboratories, Hercules, CA). The blots were scanned using the Biophotonics system (Biophotonics Corp., Ann Arbor, MI). The band intensity was evaluated using the Intelligent Quantifier software (Bio Image, Ann Arbor, MI). The overexpression of eIF4E and TLK1B was quantified as x-fold over the samples of benign tissue from noncancer specimens run concurrently on the gel.

### Analysis of TMAs

The first TMA (TMA1) was constructed to optimize antibody dilutions. The second TMA (TMA2) was designed with triplicate specimens to analyze intra-individual variability. In this regard, three separate plugs from each patient were taken from each original block and re-imbedded into TMA2. Replicate breast tumor specimens were analyzed for plug-to-plug reproducibility by staining the TMAs immunohistochemically and quantitating them using the ARIOL imaging system (described below). The third TMA (TMA3) was designed to compare eIF4E to its downstream effector proteins using a larger set of breast cancer specimens.

### ARIOL Imaging

The ARIOL imaging system (Genetix, San Jose, CA) was used to quantify antibody staining of the TMAs. The specimens were scanned at a low resolution (1.25×) and high resolution (20×) using Olympus BX 61 microscope with an automated platform (Prior). The slides were loaded in the automated slide loader (Applied Imaging SL 50). The images with high resolution were used for training and quantification purpose. The system was trained to select the stained and unstained cells/nuclei by the color of staining and shape of nuclei such that brown staining was considered positive and blue staining was considered negative. The number of cells/nuclei stained was calculated and represented as percentage of total cells/nuclei stained positively. By measuring both immunostaining intensity and percentage, data obtained are reproducible, objective measurements of immunoreactivity. Because standardizing IHC, from the fixation of tissues to the analysis of IHC results is critical, all immunohistochemistry data were normalized to cytokeratin. To control for the variability in tumor cellularity from one patient to another, and to also control for variations in the number of tumor cells at different TMA spots (intra-tumoral variations), the number of epithelial (tumor) cells present at each TMA spot as highlighted by expression of cytokeratin 7, was used for normalization of each protein expression studied [26]. For each protein, a score was generated based on the area with and the intensity of the brown staining reaction. The scores were then exported to an Excel spreadsheet for analysis. A normalized value was calculated for each protein by dividing the recorded score for the protein by the recorded score for cytokeratin 7 at the corresponding spot.

### ER, PR, HER-2/neu analysis

Immunohistochemical staining for estrogen receptor (ER), progesterone receptor (PR), and HER-2/neu was performed using automated processing and staining technology (BenchMark XT IHC/ISH, Ventana). Processes included deparaffinization, pretreatment, antibody incubation, counterstaining, and coverslipping. Levels of membranous/cytoplasmic immunostaining for Her-2/neu, were scored using an automated cellular image analysis system (ACIS) (Clarient, San Juan Capistrano). Values less than 1.9 are interpreted as negative and values ≥ 2.0 are interpreted as positive for HER-2/neu over-expression. Nuclear ER and PR expression was assessed using the ACIS; both the quantitative intensity of expression and percentage of cells showing positive expression were noted.

### Statistical analysis

Intra-individual coefficient of variations (CV) was calculated as ratio of standard deviation over mean × 100. The mean CV% and SD of CV for each marker was also added. The correlation among the expression levels of eIF4E, c-Myc, cyclin D1, ODC, TLK1B, VEGF, ER, PR, and HER-2/neu were calculated by the Spearman rank correlation method. These correlation coefficients were test against 0. All two-sided p-values < 0.05 were considered as statistically significant. The strength of correlation among the markers were classified as strong, moderate and weak for the correlation coefficient > 0.8, 0.4–0.8, and < 0.4 respectively. The statistical software used for the current study was SAS 9.1.3. SAS Institute Inc., Cary, NC.

## Results

### Construction and analysis of TMAs

**Figure 1:**
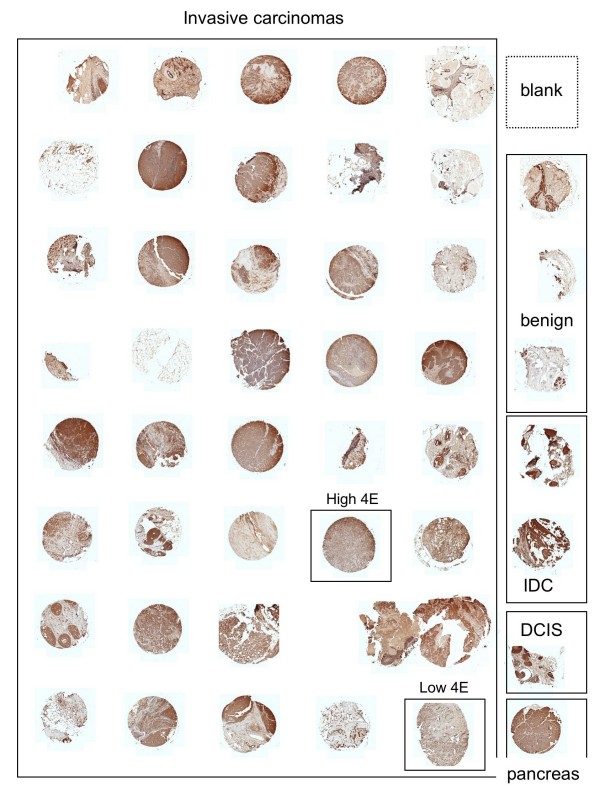
**Low magnification (100 ×) of human breast cancer specimens in TMA3 stained immunohistochemically for ODC**. Boxes indicate specimen type. The specimens marked "low 4E" and "high 4E" are also shown in Figure 3. The top right corner of the TMA was left blank, and a pancreas specimen was placed in the lower right hand corner of the TMA in order to verify proper orientation. IDC, intraductal carcinoma; DCIS, ductal carcinoma in situ.

**Figure 2:**
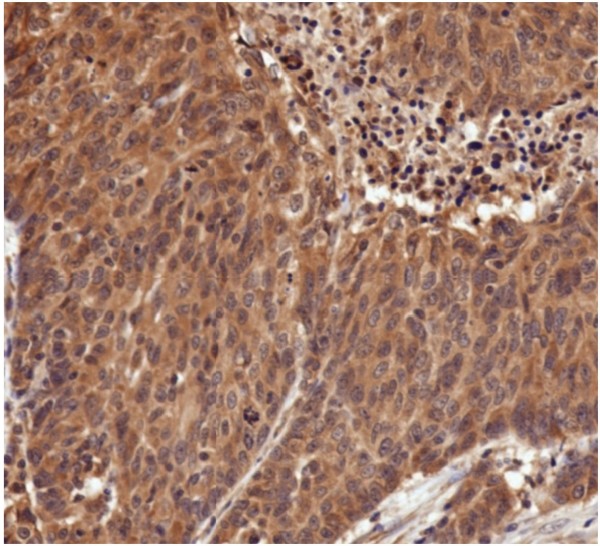
**High magnification (400 ×) of human breast cancer specimen from TMA3 stained immunohistochemically for ODC**. Note the predominantly cytosolic staining of ODC, whereas the nuclei were counterstained blue.

The first TMA was constructed in order to optimize the immunohistochemical staining techniques and to train the ARIOL imaging system. The criteria for successful staining included appropriate staining to the subcellular compartment, lack of staining in the absence of primary antibody, increase in staining when higher concentrations of primary antibodies were used, low staining in non-epithelial derived tissue (such as stroma or fat), and low staining in the negative controls (benign tissue). An example of the construction of TMA3 is shown in Figure 1. The ARIOL system first images the entire slide to show each plug. Higher resolution images can be made by zooming in on each plug. As shown in Figure 2, the ARIOL system can be trained to distinguish between cytoplasmic and nuclear staining. For example, ODC typically stains in the cytoplasm, leaving the counter-stained nuclei predominantly blue (Figure 2). The computational software can then scan and analyze each plug for positive staining.

### Intra-individual coefficients of variances

Once these conditions were established, the second TMA was constructed using replicate plugs in order to verify the plug-to-plug consistency for each protein. The intra-individual coefficients of variances (CV%) for eIF4E, c-Myc, cyclin D1, ODC, TLK1B and VEGF were used as a measure of plug-to-plug reproducibility (Table 1). The overall CV% (means ± SE) was 35.8 ± 5.3%. The range of CV% was 25.2 ± 6.1% (VEGF) up to 55.9 ± 14.2% (cyclin D1). Since the TMAs can have up to 48 specimens, future TMAs could be made by using up to 48 individual, 24 duplicate, or 16 triplicate specimens (minus appropriate controls). Based on these CV% results, TMA3 was created using individual specimens, because we felt that the overall CV% was reasonable and that more power could be gained by analyzing a larger number of individual specimens.

**Table 1:** Intra-individual Coefficients of Variance for TMA2 (CV%)^a^

	Mean IOD	SD IOD	Mean CV%	SD CV%	SE CV%	n
1. eIF4E	62.7	26.2	26.4	24.5	7.8	10
2. c-Myc	68.1	23.3	28.1	16.1	4.9	11
3. Cyclin D1	51.2	32.5	55.9	45.1	14.2	10
4. ODC	55.2	23.4	30.7	27.2	8.6	10
5. TLK1B	38.9	26.3	46.9	38.5	11.6	11
6. VEGF	24.8	15.3	25.2	18.4	6.1	9

						
Overall			35.5	12.8	5.2	6

^a^Intra-individual coefficient of variations (CV) was calculated as ratio. of standard deviation over mean × 100. The mean CV% and SD of CV for each marker was also added. The N's were added up in Table 1 as the number of replicate cases. Only those specimens in which 2–3 plugs could be analyzed are listed. So, in TMA2, there were up to 12 different cases, but only those that resulted in duplicate or triplicate plugs were analyzed for CV%. The overall mean and SD for integrated optical density (IOD) for each protein is also listed.

### TMA-IHC analysis: Correlation of eIF4E with downstream effector proteins

**Figure 3:**
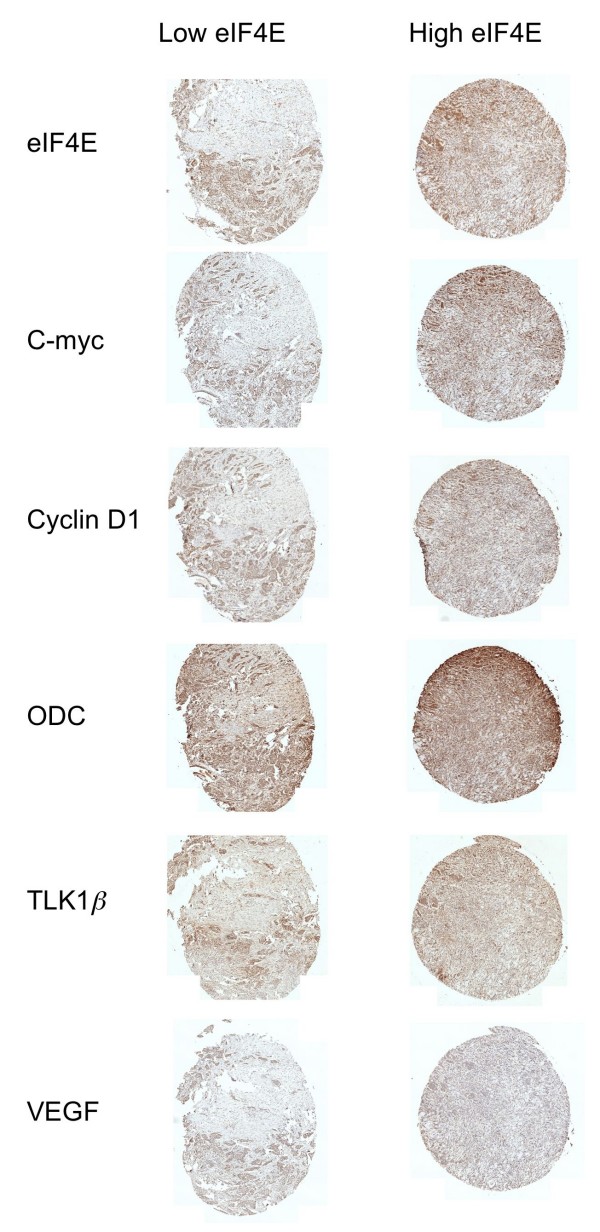
**Representative example of human breast cancer specimens from TMA3 that expressed either low (left panel) or high (right panel) eIF4E**. Matching specimens from the same patient are shown for c-Myc, cyclin D1, ODC, TLK1B, and VEGF (200 × magnification).

**Figure 4:**
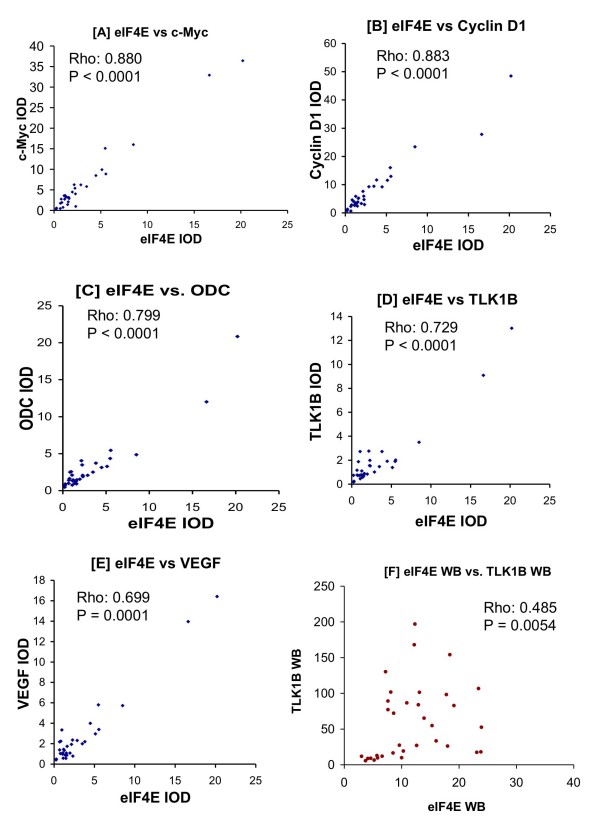
**Correlation of immunohistochemical expression of eIF4E vs c-Myc [A], cyclin D1 [B], ODC [C], TLK1B [D], VEGF [E] from TMA3**. Figures represent the integrated optical density (IOD) of immunohistochemical staining intensity normalized to cytokeratin. Protein expression of eIF4E and TLK1B were also compared by western blot analysis [F], in which values represent expression of eIF4E and TLK1B as fold- over benign. All comparisons were done using Spearman's rank correlation. Rho- and p- values for each comparison are displayed in each panel.

In TMA3, eIF4E expression levels correlated strongly with the downstream effector proteins, c-Myc, cyclin D1, ODC, TLK1B, and VEGF (Figures 3, 4). In Figure 3, we show a set of human breast carcinoma specimens from TMA3 that were either low or high for eIF4E (as measured by IHC). Their positions on TMA3 are marked in Figure 1. Then, the IHCs for the same specimens are also shown for the downstream effector proteins. Generally, specimens that possessed high eIF4E protein expression also exhibited high expression of c-Myc, cyclin D1, TLK1B, VEGF, and ODC. Likewise, specimens that expressed low amounts of eIF4E protein also expressed low amounts of c-Myc, cyclin D1, TLK1B, VEGF, and ODC. The rho values for eIF4E compared to c-Myc, cyclin D1, TLK1B, VEGF, and ODC were 0.880, 0.863, 0.729, 0.699, and 0.799 respectively, and all these comparisons were statistically significant at p ≤ 0.0001 (Figure 4A–E).

### Western blot analysis: Correlation of eIF4E with TLK1B

We have previously shown by western blot analysis that the expression of eIF4E correlated with that of TLK1B [23]. As further validation of our TMA results, we also compared eIF4E with TLK1B using the corresponding fresh-frozen specimens from the same tumors as those used for TMA3 (Figure 4F). Due to limited amounts of fresh-frozen specimens, the other proteins were not analyzed. Protein expressions of eIF4E to TLK1B were positively correlated (rho value 0.485, p value 0.0054).

### Non-correlation to independent markers

We have previously demonstrated that western blot analysis of eIF4E did not correlate with node status, ER, PR, or HER-2/neu [18,19]. In the current study, expression of eIF4E (by both TMA-IHC and western blot) was also compared to ER, PR, and HER-2/neu expression. There was no correlation of eIF4E on TMA3 with any of these independent markers by either TMA-IHC or western blot analysis of eIF4E (Table 2).

**Table 2:** Lack of correlation of ER, PR, or HER-2/neu with eIF4E

		95% Confidence Interval			
				
	Rho Value	Lower	Upper	n	P
*TMA expression of eIF4E*^a^					
eIF4E and ER	-0.137	-0.469	0.228	31	0.452
eIF4E and PR	-0.069	-0.413	0.293	31	0.707
eIF4E and HER-2/neu	-0.013	-0.406	0.384	25	0.949
*Western blot expression of eIF4E*^b^					
eIF4E and ER	-0.192	-0.479	0.132	39	0.237
eIF4E and PR	-0.295	-0.558	0.023	39	0.069
eIF4E and HER-2/neu	-0.143	-0.469	0.216	32	0.425

^a ^For the first three rows, comparisons were made of immunohistochemical staining of each protein normalized to cytokeratin to ER, PR, and HER-2/neu. ^b^Last three rows, comparison of protein expression of eIF4E assayed by western blot (fold- over benign) to ER, PR, and HER-2/neu. All comparisons were done using Spearman's Rank Correlation.

## Discussion

In the current study, we have analyzed the expression of eIF4E along with 5 of its downstream effector proteins in human breast carcinoma specimens using immunohistochemical analysis of TMAs. The downstream effector proteins c-Myc, cyclin D1, ODC, TLK-1B, and VEGF were positively correlated with eIF4E when analyzed by IHC. As shown previously [27], western blot analysis of eIF4E correlated with TLK1B protein expression. At the same time, eIF4E expression (determined by both western blot and TMA-IHC) did not correlate with ER, PR or HER-2/neu. Consistent with these results, the lack of correlation of eIF4E (detected by western blot) with ER, PR, and HER-2/neu was previously reported [18,19]. Our results confirm and extend the results previously described by Yang and colleagues [20]. In their study, which utilized a multi-tumor TMA from TARP , they found eIF4E, VEGF, and cyclin D1 were elevated in breast tumors compared to combined normal tissues [20]. The authors also found that eIF4E levels were moderately correlated with VEGF and cyclin D1 expression in breast (Spearman's rank correlation) [20]. Among the major differences between the two studies: this study focused solely on breast cancer, and included validation of western blot *and *IHC analysis of the same samples. We also verified coefficients of variance to demonstrate plug-to-plug reproducibility. Furthermore, we examined a broader range of downstream proteins, and included more negative controls. Also, we used the ARIOL imaging system whereas they used ACIS. The strength of these two studies supports the idea that IHC can be used in a TMA format for evaluating critical oncogenic proteins.

In comparing western blot to IHC, there are advantages and disadvantages to both procedures. One advantage to western blot, traditionally, is that it has provided a greater dynamic range for quantitation than IHC. This is especially true, historically, as IHC has been semi-quantitative and subject to scoring methods. However, with the wider availability of IHC quantitation systems such as ARIOL, IHC has become more quantitative. This also provides potential standardization between different research institutions. The use of TMAs rather than individual IHCs for each specimen also provides institutions the ability to analyze a larger set of specimens at a time using similar staining and quantitation procedures. Another advantage to western blot, however, is that the molecular weights of the proteins can be estimated based on the molecular weight standards that are also resolved on the gel. This is particularly important if the antibodies exhibit non-specific staining. The protein of interest can be isolated from the non-specific staining and quantitated. The best way to overcome the problem of non-specific staining in IHC is to use the most specific antibodies available and to optimize the dilutions of antibodies and other staining conditions. Comparisons of positive and negative controls also help confirm specificity. One disadvantage to western blot is that unless specimens were prepared by isolation of specific cells through differential centrifugation or density gradients, they typically contain a heterogeneous mixture of cells (e.g., stromal component, adipocytes, epithelial cells, necrotic tissue, vascular tissue, etc.) and may not distinguish between the different compartments of the cell. With the ARIOL imaging system, different regions of tissue can be selected and quantitated, so as to avoid sections that contain non-regions of interest. Furthermore, ARIOL also possesses the training capability to select nuclear vs. cytoplasmic staining. Also, large amounts of precious tissue are required for western blots, which may not be readily available. TMAs or IHC require less sample, and archived specimens can be used for a longer follow-up period. An average of 30–40 serial sections can be cut from one of our TMAs, such that multiple comparisons can be drawn among different proteins of interest. For these reasons, we believe that TMAs will provide a reasonable method for analyzing large numbers of specimens.

It has been shown that eIF4E is an independent prognostic factor in breast cancer [18]. We had selected tumor samples that showed a wide range of eIF4E protein expression by western blot which was significantly higher than the normal tissues. The TMA staining showed that 4E was elevated in breast tissues compared to the normal tissues. Over-expression of eIF4E leads to the translation of structured 5' UTR mRNAs which include c-Myc, cyclin D1, ODC, TLK1B and VEGF. These proteins have been studied individually in breast cancer patients. The results of the current study have shown that when eIF4E was elevated there was a corresponding rise in the protein expression of c-Myc, cyclin D1, ODC, TLK1B and VEGF. Thus eIF4E modulates the expression of the downstream effector proteins that regulate processes up regulated in cancer cells like the cell cycle, survival and cell growth. On the other hand, previous results using western blot analysis of eIF4E demonstrated that it did not correlate with node status, ER, PR, or HER-2/neu expression [18,19]. As a negative control for our current study, we also showed that IHC analysis of eIF4E on TMA3 also did not correlate with ER, PR, or HER-2/neu. Western blot analysis of eIF4E from the corresponding samples showed similar results.

## Conclusion

To our knowledge, this is the first time that a correlation has been made in a single study between eIF4E, c-Myc, cyclin D1, ODC, TLK1B and VEGF. Since the samples were obtained from a geographical area in which patients typically present with advanced stage breast cancer [28], this study has shown the major oncoproteins that are upregulated in this population. The hospital also possesses the clinical information as well as the outcome of these patients. This study becomes more relevant when we can correlate the results from the TMA study to the clinical outcome as we follow up with these patients. In conclusion, eIF4E preferentially upregulates gene products that are involved in worse clinical outcome in breast cancer, head and neck cancer, and others. To study each downstream effector protein by western blot using fresh-frozen tissue is cumbersome and simply not feasible for many reasons, including long-term follow-up. There is an urgent need for clinicians to be able to examine a set of biomarkers such as eIF4E and downstream effector molecules in order to set a current standard for prognosis.

## Abbreviations

ER: estrogen receptor; eIF4E: eukaryotic initiation factor 4E; CV: intra-individual coefficients of variance; HER-2/neu: human epidermal growth factor receptor-2; IHC: immunohistochemical analysis; IOD: integrated optical density; ODC: ornithine decarboxylase; PR: progesterone receptor; TLK1B: tousled-like kinase 1B; TMA: tissue microarray; 5'-UTR: 5'-untranslated regions; VEGF: vascular endothelial growth factor; WB: western blot.

## Competing interests

The authors declare that they have no competing interests.

## Authors' contributions

The ARIOL imaging and analyses were done by JT, MS, M L-N, and MU. PA, JC, JC, and BL designed and constructed the TMAs. Western blots were done by MS and CM. Immunohistochemical staining of the TMAs was performed by CM and PK. Analysis of Her2/Neu, ER, and PR was performed by ML-N. Statistical analysis was done by RS. QC and JM assisted with immunohistochemical staining, design, and interpretation of the study. Overall supervision, planning and preparation of the manuscript were completed by HK and BL.
