# Tissue microarray analysis of eIF4E and its downstream effector proteins in human breast cancer

**DOI:** 10.1186/1756-9966-28-54

**Published:** 2009-04-24

**Authors:** Heather E Kleiner, Prasad Krishnan, Jesse Tubbs, Mark Smith, Carol Meschonat, Runhua Shi, Mary Lowery-Nordberg, Patrick Adegboyega, Marcia Unger, James Cardelli, Quyen Chu, J Michael Mathis, John Clifford, Arrigo De Benedetti, Benjamin DL Li

**Affiliations:** 1 Dept of Pharmacology, Toxicology, and Neuroscience, Breast Cancer Focus Group, Feist-Weiller Cancer Center, Shreveport & LSUHSC-Shreveport, Louisiana, LA 71130, USA; 2 Dept Surgical Oncology, Breast Cancer Focus Group, Feist-Weiller Cancer Center, Shreveport & LSUHSC-Shreveport, Louisiana, LA 71130, USA; 3 Dept of Medicine, Breast Cancer Focus Group, Feist-Weiller Cancer Center, Shreveport & LSUHSC-Shreveport, Louisiana, LA 71130, USA; 4 Dept of Pathology, Breast Cancer Focus Group, Feist-Weiller Cancer Center, Shreveport & LSUHSC-Shreveport, Louisiana, LA 71130, USA; 5 Dept of Microbiology & Immunology, Breast Cancer Focus Group, Feist-Weiller Cancer Center, Shreveport & LSUHSC-Shreveport, Louisiana, LA 71130, USA; 6 Dept of Cellular Biology & Anatomy, Breast Cancer Focus Group, Feist-Weiller Cancer Center, Shreveport & LSUHSC-Shreveport, Louisiana, LA 71130, USA; 7 Dept of Biochemistry & Molecular Biology, Breast Cancer Focus Group, Feist-Weiller Cancer Center, Shreveport & LSUHSC-Shreveport, Louisiana, LA 71130, USA

## Abstract

Correction to Kleiner HE, Krishnan P, Tubbs J, Smith M, Meschonat C, Shi R, Lowery-Nordberg M, Adegboyega P, Unger M, Cardelli J *et al*: Tissue microarray analysis of eIF4E and its downstream effector proteins in human breast cancer. *J Exp Clin Cancer Res *2009, 28:5.

## Correction

After publication of the work [1], we noticed that we inadvertently failed to acknowledge an additional funding source. HK was supported by a National Cancer Institute grant 1K22CA102005-01A2. The content is solely the responsibility of the authors and does not necessarily represent the official views of the National Cancer Institute or the National Institutes of Health.
